# Acetate and Bicarbonate Assimilation and Metabolite Formation in *Chlamydomonas reinhardtii*: A ^13^C-NMR Study

**DOI:** 10.1371/journal.pone.0106457

**Published:** 2014-09-10

**Authors:** Himanshu Singh, Manish R. Shukla, Kandala V. R. Chary, Basuthkar J. Rao

**Affiliations:** 1 Department of Chemical, Tata Institute of Fundamental Research, Mumbai, India; 2 Department of Biological Sciences, Tata Institute of Fundamental Research, Mumbai, India; 3 Tata Institute of Fundamental Research, Center for Interdisciplinary Sciences, Hyderabad, India; University of Hyderabad, India

## Abstract

Cellular metabolite analyses by ^13^C-NMR showed that *C. reinhardtii* cells assimilate acetate at a faster rate in heterotrophy than in mixotrophy. While heterotrophic cells produced bicarbonate and CO_2_^aq^, mixotrophy cells produced bicarbonate alone as predominant metabolite. Experiments with singly ^13^C-labelled acetate (^13^CH_3_-COOH or CH_3_-^13^COOH) supported that both the ^13^C nuclei give rise to bicarbonate and CO_2_^aq^. The observed metabolite(s) upon further incubation led to the production of starch and triacylglycerol (TAG) in mixotrophy, whereas in heterotrophy the TAG production was minimal with substantial accumulation of glycerol and starch. Prolonged incubation up to eight days, without the addition of fresh acetate, led to an increased TAG production at the expense of bicarbonate, akin to that of nitrogen-starvation. However, such TAG production was substantially high in mixotrophy as compared to that in heterotrophy. Addition of mitochondrial un-coupler blocked the formation of bicarbonate and CO_2_^aq^ in heterotrophic cells, even though acetate uptake ensued. Addition of PSII-inhibitor to mixotrophic cells resulted in partial conversion of bicarbonate into CO_2_^aq^, which were found to be in equilibrium. In an independent experiment, we have monitored assimilation of bicarbonate via photoautotrophy and found that the cells indeed produce starch and TAG at a much faster rate as compared to that in mixotrophy and heterotrophy. Further, we noticed that the accumulation of starch is relatively more as compared to TAG. Based on these observations, we suggest that acetate assimilation in *C. reinhardtii* does not directly lead to TAG formation but via bicarbonate/CO_2_^aq^ pathways. Photoautotrophic mode is found to be the best growth condition for the production of starch and TAG and starch in *C. reinhardtii.*

## Introduction

In the genus *Chlamydomonas,* the *C. reinhardtii* species has attracted attention over the last several decades due to the ease of generating photosynthetic mutants that can be maintained in dark on acetate as the sole carbon source [1]. Although, it is generally assumed that the acetate assimilation takes place primarily through the glyoxylate and TCA cycle pathways, a systematic study of the assimilation and its metabolic regulation in different conditions is still lacking. In addition, the integration of acetate metabolism across its four active metabolic compartments, namely, the chloroplast, mitochondria, glyoxysomes and the cytosol is not well understood [2]. The metabolism in *C. reinhardtii* must be flexible due to its ability to dynamically transition between photoautotrophic, solely acetate-based heterotrophic and mixotrophic states [3]. The metabolic switches that operate as cells traverse between photoautotrophy, mixotrophy and heterotrophy and back are far from clear.

It is believed that acetate is converted to acetyl-CoA by the enzyme acetyl-CoA synthase or by a combination of the enzymes, acetate kinase and phosphate acyl transferase [4], [5]. Acetyl-CoA is assimilated predominantly in the mitochondria and glyoxysomes of *C. reinhardtii* cells where in the acetate carbons get recruited into the carbon intermediates of the glyoxylate and TCA cycle. It is established that glyoxylate cycle is preferred over TCA cycle by *C. reinhardtii* cells in light, whereas the preference is reversed in dark [6]. As a relatively minor route, acetyl-CoA is also conjectured to get assimilated in the chloroplast compartment and in cytoplasm leading to both the amino-acid and the fatty-acid biosynthesis.

In this backdrop, we set out to study the carbon assimilation in *C. reinhardtii* by using *in-vivo* NMR spectroscopy. *In-vivo* NMR spectroscopy allows the generation of time-resolved measurements, when cells are metabolically active [7]. *In-vivo* NMR provides information on metabolomics, where sample-processing artifacts are minimized. However, sensitivity of this technique is not high, and hence, one can only detect metabolites that are close to or higher than milli-molar levels [8]–[10]. However, high-field NMR spectrometers (>600 MHz) equipped with a cryogenically cooled probe could dramatically enhance the sensitivity [11]. The spectral signature arising from predominant metabolite signals can be further enhanced using ^13^C-labeled compounds, which could provide new insights into the metabolic flux of the organism [12].

In this study, we aimed to understand the *in-vivo* carbon (acetate and bicarbonate) assimilation and metabolism in *C. reinhardtii* during light (mixotrophic/autotrophic) and dark (heterotrophic) phases of growth as well as in nutritionally starved state of the cells. We set out to assess major differences in metabolite profiles in these three growth conditions. Acetate assimilation led to the accumulation of glycerol in dark, which was converted to triacylglycerol (TAG) in light, while starch was observed under both conditions. Further metabolic changes took place, when the cells were incubated longer up to eight days without the addition of fresh acetate, where in bicarbonate and CO_2_^aq^ were routed towards TAG production, underscoring the fact that acetate assimilation functions via inorganic carbon pathway rather than by direct incorporation. In contrast, photoautotrophic conditions showed much more rapid conversion of inorganic carbon (Ci: bicarbonate plus CO_2_^aq^) towards starch and lipid production than in hetero/mixotrophic modes.

This study provides the initial account of comparative analyses of carbon uptake and assimilation *in-vivo*, in real time. We observed that acetate assimilation in *C. reinhardtii* does not directly lead to TAG formation but via bicarbonate/CO_2_^aq^ pathways. Further, the photoautotrophic mode is found to be the best growth condition for the production of starch and TAG in *C. reinhardtii.*

## Results

### Acetate assimilation during heterotrophy

**Figure 1:**
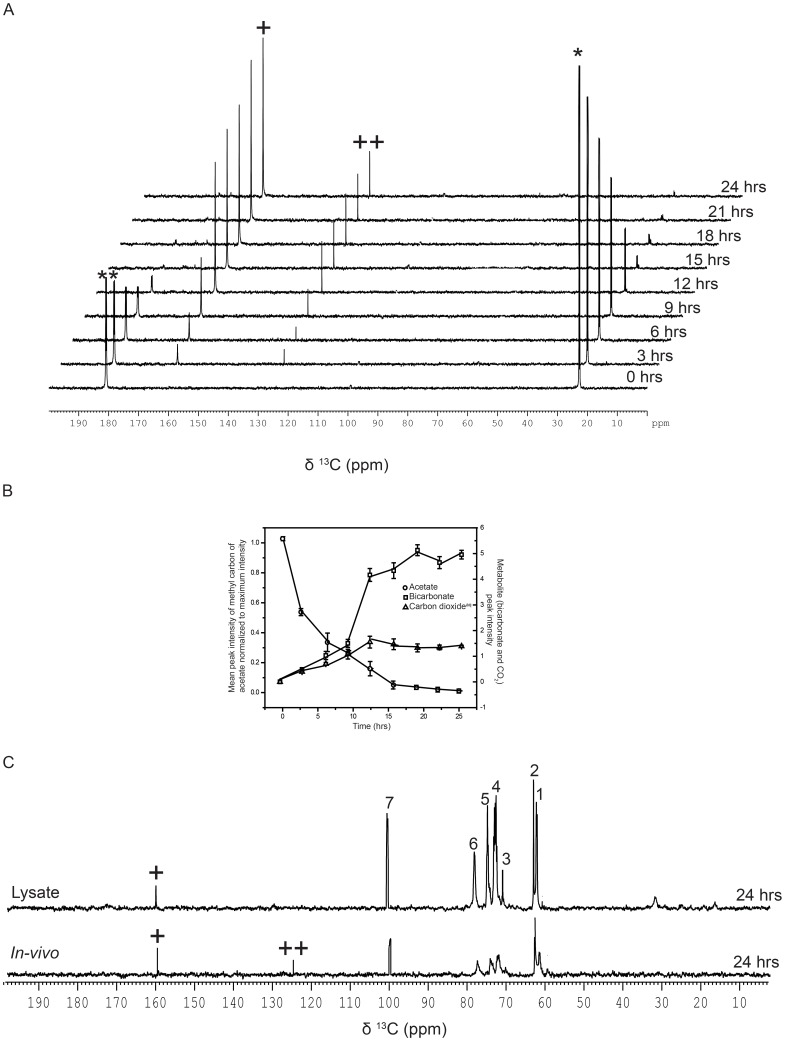
Assimilation kinetics of [1, 2-^13^C]-acetate in heterotrophy. (A) Assimilation kinetics of acetate by *C. reinhardtii*, as studied by recording proton decoupled 1D^13^C-NMR spectra, during heterotrophic growth at different time points after adding the [1, 2-^13^C]-acetate to the TP growth medium (see Fig. S1). The spectra from the same experiment are stacked for representation. Peaks at 125.48 and 161.01 ppm show CO_2_^aq^ and bicarbonate, respectively. (B) Quantitative analyses of [^13^C]-methyl-peak intensity of acetate and metabolites (bicarbonate and CO_2_^aq^) at different time intervals during heterotrophy. Peak intensities (area under the peaks) were normalized with respect to the 0 hr data for acetate. Values are mean of three independent experiments ± SD. (C) Proton decoupled 1D [^13^C]-NMR spectra recorded on 700 MHz with TXO probe optimized for direct [^13^C]-detection at 298 K with heterotrophic cells grown in dark for 24 hrs in TP growth medium containing [1, 2-^13^C]-acetate. The peaks 1 and 3 detected at 61.93 and 72.48 ppm, respectively, represent carbons from glycerol (J_CC_ = 41 Hz and J_CH_ = 142 Hz) while peaks 2, 4, 5, 6 and 7 at 62.97, 72.88, 74.79, 78.32 and 101.06 ppm, respectively, represent carbons from starch. (The signs * and ** represent methyl and carboxyl peaks, respectively, of [1, 2-^13^C]-acetic acid, and the signs^+^and^++^represent bicarbonate and CO_2_^aq^ peaks, respectively).

Assimilation of [1, 2-^13^C]-acetate was followed during the heterotrophic growth conditions as illustrated in Fig. S1. The first 1D ^13^C NMR spectrum of Fig. 1A showed two doublets, at 23.96 and 182.16 ppm, corresponding to the methyl and the carboxylic ^13^C nuclei, respectively. No other spectral signature was seen in the spectrum that could be attributed to as arising from the unlabeled acetate added to the pre-culture. For clarity, we have expanded the individual doublets, highlighting the ^13^C-^13^C coupling of ∼52.9 Hz, in the proton decoupled 1D ^13^C-NMR spectrum of [1, 2-^13^C]-acetate (Fig. S2). As shown in Fig. 1A, the intensity of both the peaks expectedly decreased as a function of time with acetate uptake. The spectra in Fig. 1A, collected from the same experiment are arranged in a “stacked format” for better visual rendering. As shown in Fig. 1A and 1B, in about 18 hrs of incubation, most of the acetate signal got depleted due to the cellular assimilation. The uptake rates of methyl and carboxyl-^13^C spins, as measured by their decreasing individual peak intensities, were similar. Quantitative analyses of acetate uptake kinetics and the concomitant accumulation of metabolites are shown in Fig. 1B. The uptake of acetate resulted in concomitant showing up of two metabolites in about three hours of incubation, whose intensity increased as a function of incubation time. These two predominant metabolites observed at 125.48 and 161.01 ppm were assigned as arising from ^13^CO_2_^aq^ and bicarbonate, respectively, by comparing these values with respective standard values (Fig. 1A, S3A and S3B). Detection of ^13^CO_2_^aq^ in TAP medium (pH 7.0) could be due to the metabolic processes mediated by enzymes like carbonic anhydrases in the cell and certainly not due to the chemical conversion of bicarbonate to CO_2_^aq^ [13] (see Fig. S3C), which occurs only at an acidic pH [14]. To justify this conclusion, in an independent experiment, ^13^C-bicarbonate was subjected to varying pH conditions, where in we detected ^13^CO_2_ spectral signature with the concomitant depletion of ^13^C-bicarbonate only at lower pH (Fig. S3C).

To estimate residual ^13^C-acetate, if any, in the cell-free medium, cells were pelleted down and suspended in an unlabeled medium (TAP, pH 7.0), followed it by NMR analyses of label in both cell free supernatant (Fig. S4A) and pellet fractions (Fig. S4B). As shown in the stacked plots of Fig. S4A and Fig. S4B, no acetate signatures were detectable in both the fractions after 12 hrs of incubation, indicating the complete conversion of acetate into metabolites. Analyses of supernatant and pellet fractions revealed reduced levels of bicarbonate in both fractions (Fig. S4A and S4B). In contrast, CO_2_^aq^ signal was largely seen in the pellet sample (Fig. S4B).

Further, to identify additional metabolites, if any, we used a high-sensitive triple-resonance observe (TXO) probe, which was specifically designed for ^13^C-direct detection with an enhanced signal-to-noise (S/N) ratio, on a Bruker Avance 700 MHz NMR spectrometer. To further enhance the S/N ratio, the culture sample was pelleted down and re-suspended in ^2^H_2_O before recording the 1D ^13^C-NMR spectrum. This enabled us to observe several resonances (numbered 1 to 7) in the ^13^C-NMR spectrum ranging from 59–126 ppm, though with still poor S/N ratio (bottom spectrum in Fig. 1C). We then resorted to sonicate the cells and recorded the 1D ^13^C-NMR spectrum with the lysate (top spectrum in Fig. 1C). This resulted in a dramatic enhancement of the S/N ratio. We could assign the peaks numbered 1 and 3 (at 61.93 and 72.48 ppm, respectively) to glycerol (Fig. S5A) and the peaks numbered 2, 4, 5, 6 and 7 (at 62.97, 72.88, 74.79, 78.32 and 101.06 ppm, respectively) to starch (Fig. S5B and 1C) by comparing their chemical shifts with respective standard values. Thus, the ^13^C-NMR spectrum of the cell lysate recorded with the use of TXO probe helped us to identify additional metabolites, such as starch and glycerol.

**Figure 2:**
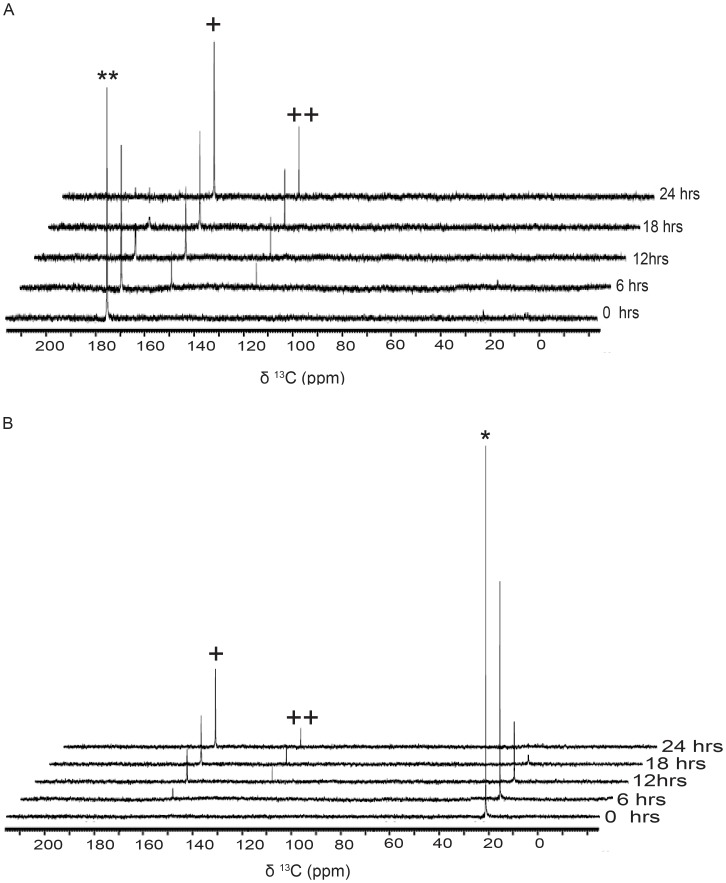
Assimilation kinetics of Carboxyl and Methyl [^13^C]-carbons of acetate in heterotrophic cells. (A) Assimilation kinetics of acetate by *C. reinhardtii*, as studied by recording 1D [^13^C]-NMR spectra at different time points during heterotrophy following the addition of [1-^13^C]-acetate (where carboxyl carbon is labeled) in the TP medium. (The sign ** Represents carboxyl peak of [1, 2-^13^C]-acetic acid, and the signs^+^and^++^represent bicarbonate and CO_2_^aq^ peaks respectively). (B) Assimilation kinetics of acetate by *C. reinhardtii*, as studied by recording 1D proton decoupled [^13^C]-NMR spectra at different time points during heterotrophy following the addition of [2-^13^C]-acetate (where in the methyl carbon is labeled) in the TP medium. (The sign * represents methyl peak of [1, 2-^13^C]-acetic acid, and the signs^+^and^++^represent bicarbonate and CO_2_^aq^ peaks, respectively).

In order to assess whether the labeled ^13^C spins in both the metabolites, namely bicarbonate and CO_2_^aq^, arose from both the ^13^C spins present in the [1, 2-^13^C]-acetate precursor or not, we performed metabolite analyses with singly-^13^C-labeled acetate (labeled at position 1 or 2) as the sole source of carbon. The experiments revealed that both the metabolites indeed arose from both sources of ^13^C-spins present in the labeled acetate (Fig. 2A and 2B). However, the conversion of carboxyl-^13^C was measurably faster and greater towards net Ci released per unit acetate in the cells, as compared to the methyl-^13^C (Fig. S6). We discuss this metabolic asymmetry later in the discussion. The rate of net Ci release per acetate was constant across 24 hrs, suggesting a constant metabolic flux in the cells during this experimental regime of acetate assimilation.

### Acetate assimilation during mixotrophy

**Figure 3:**
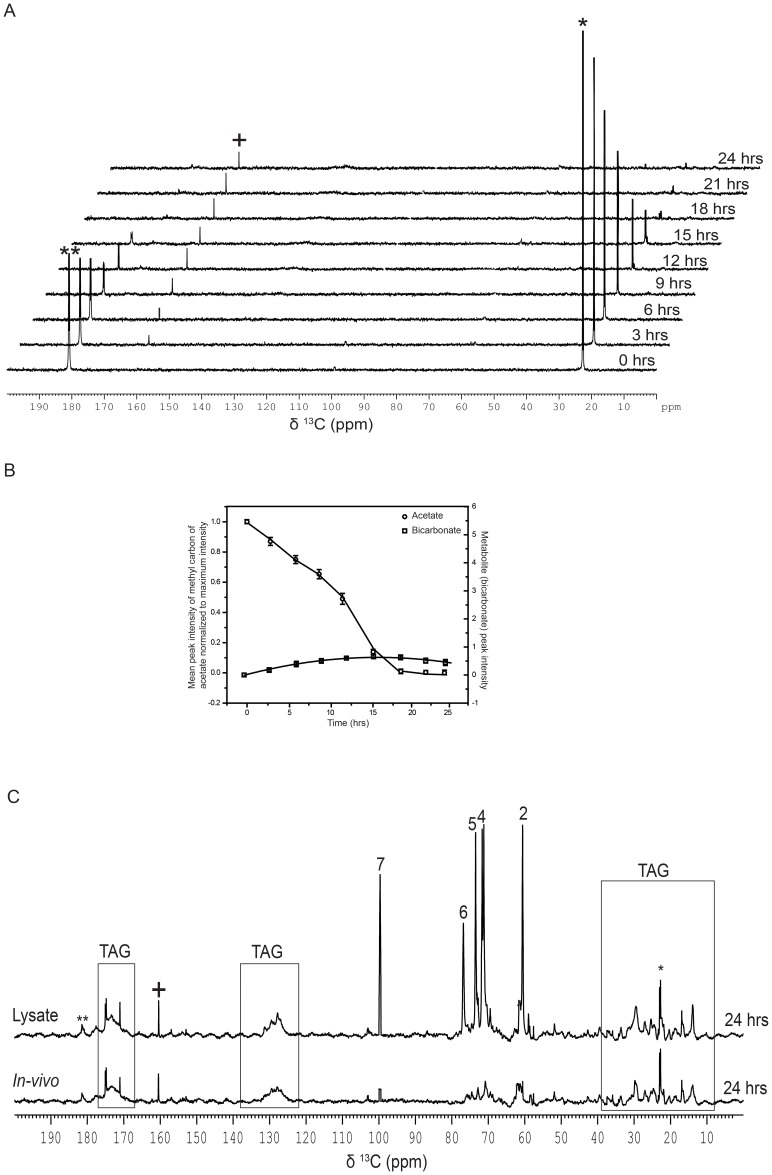
Assimilation kinetics of [1, 2-^13^C]-acetate during mixotrophy. (A) Assimilation kinetics of acetate by *C. reinhardtii*, as studied by recording proton decoupled 1D [^13^C]-NMR spectra, during mixotrophic growth at different time points following the addition of [1, 2-^13^C]-acetate to the TP growth medium. The spectra from the same experiment are stacked for representation. (The signs * and ** represent methyl and carboxyl peaks respectively of [1, 2-^13^C]-acetic acid, and the signs^+^and^++^represent bicarbonate and CO_2_^aq^peaks respectively). (B) Quantitative analyses of [^13^C]-methyl peak intensity of acetate and metabolites (bicarbonate) at different time intervals during mixotrophy. Peak intensities (area under the peaks) were normalized with respect to the 0 hr data for acetate. Values are mean of three independent experiments ± SD. (C) Proton decoupled 1D [^13^C]-NMR spectra recorded on a 700 MHz NMR spectrometer equipped with a TXO probe, optimized for direct [^13^C] detection, at 298 K of cells grown in mixotrophy for 24 hrs in TP growth medium containing [1, 2-^13^C]-acetate. Starch and lipid signals are assigned by comparison with corresponding standards (see legend Fig. S5).

Metabolic assimilation of [1, 2-^13^C]-acetate was followed during the mixotropic growth conditions as illustrated in Fig. S1. The acetate uptake by cells grown in continuous light incubation (mixotrophy) was found to be slower as compared to the cells incubated in the dark. At the end of first twelve hours of incubation, only ∼50% of ^13^C-labelled acetate was depleted during mixotrophy as compared to ∼90% depletion during heterotrophy (Fig. 3A; Compare 3B with 1B). However, with in the first 20 hrs, most of the labeled ^13^C-acetate was taken in. Concomitant with acetate depletion in the medium and in cells during light phase, we noticed a time-dependent accumulation of bicarbonate alone. Contrary to that in heterotrophy, no trace of CO_2_^aq^ signal was seen in the mixotrophy, even when the light incubation was extended upto 24 hrs (Fig. 3A). Further, even the bicarbonate signal was found to be about ten fold less intense compared to the yield observed during the heterotrophic growth condition (see Fig. 1B with 3B). Active fixation of CO_2_^aq^ must have been, at least in part, responsible for the reduced level of bicarbonate and no CO_2_^aq^ production in mixotrophic culture.

Experiments with mixotrophic culture (pelleted and re-suspended in ^2^H_2_O) using the high-sensitivity TXO probe, as described above, showed weak spectral signatures arising from both starch and TAG, which were absent during the heterotrophic growth condition (Fig. 3C). To enhance the spectral sensitivity, we sonicated the cells and recorded the 1D ^13^C-NMR spectrum with the lysate. As shown in Fig. 3C, this resulted in a dramatic enhancement of spectral signatures arising from the starch peaks (numbered 2,4, 5, 6 and 7), which could be unambiguously assigned by comparing their chemical shifts with corresponding standard values (Fig. S5B). The boxed spectral lines in Fig. 3C were identified as arising from TAG present in the culture. It is worth mentioning here that upon further incubation of the mixotrophic culture, we noticed significant enhancement in TAG spectral signatures that enabled us to unambiguously assign them by comparing again their chemical shift values with corresponding standard values (Fig. S5C and D).

**Figure 4:**
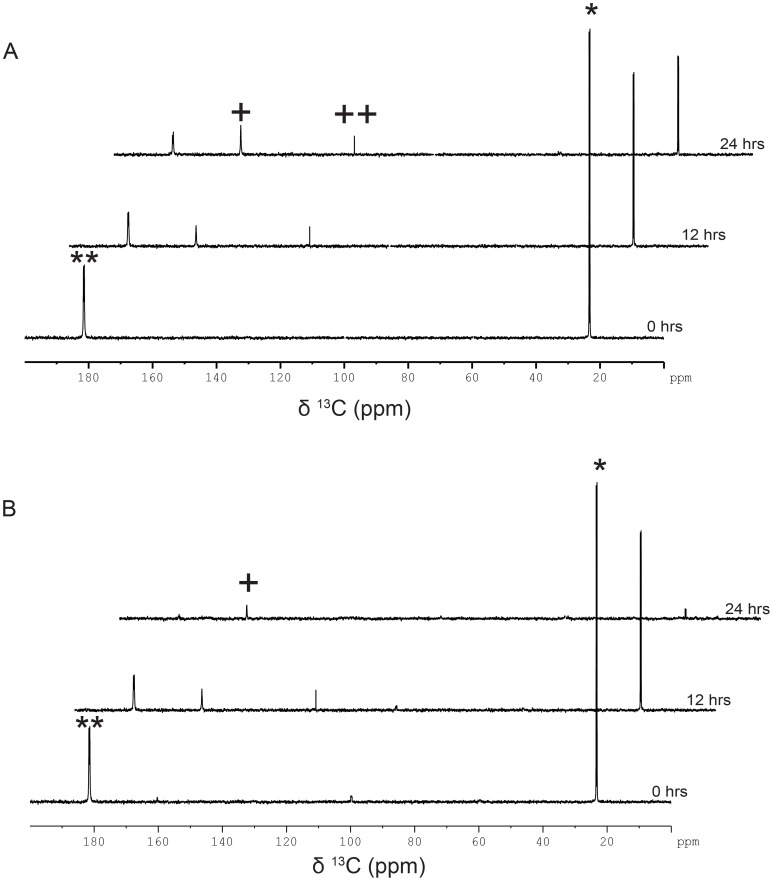
Assimilation kinetics of [1, 2-^13^C]-acetate during heterotrophy and mixotrophy in the presence of photosynthesis inhibitor atrazine. Assimilation kinetics of acetate and metabolite formation by *C. reinhardtii* during (A) heterotrophy and (B) mixotrophy following the addition of [1, 2-^13^C]-acetate to the TP medium containing atrazine (5 µM). Atrazine stock (10 mM) was prepared in water from where 5 µM atrazine was achieved. (The signs * and ** represent methyl and carboxyl peaks, respectively, of [1, 2-^13^C]-acetic acid, and the signs^+^and^++^represent bicarbonate and CO_2_^aq^ peaks, respectively).

**Figure 5:**
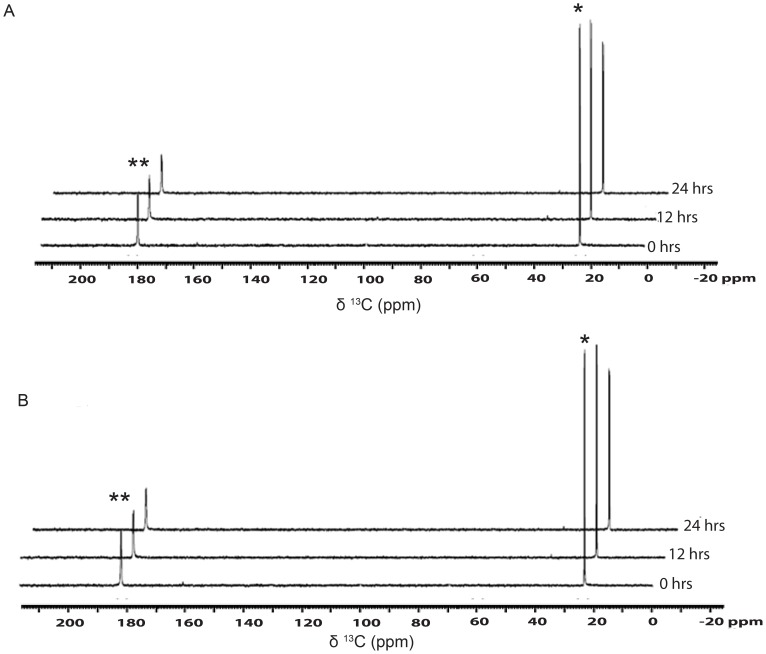
Assimilation kinetics of [1, 2-^13^C]-acetate during heterotrophy and mixotrophy in the presence of mitochondrial uncoupler CCCP. Assimilation kinetics of acetate and metabolite formation by *C. reinhardtii*, during (A) heterotrophy and (B) mixotrophy following the addition of [1, 2-^13^C]- acetate to the TP medium containing CCCP (20 µM). CCCP stock (100 mM) was prepared in DMSO from where 20 µM CCCP was achieved. (The signs * and ** represent methyl and carboxyl peaks, respectively, of [1, 2-^13^C]-acetic acid).

**Figure 6:**
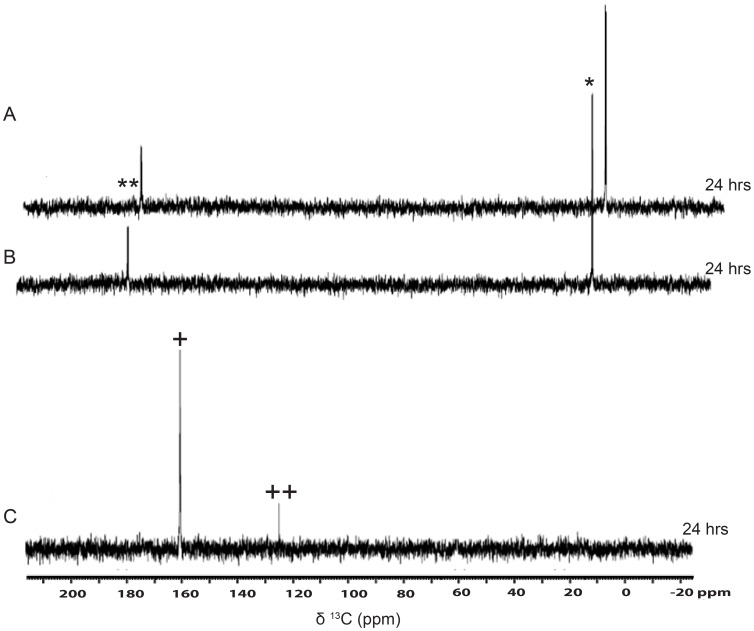
CCCP treated cells showed acetate uptake, but no conversion to metabolites in *C. reinhardtii* during heterotrophic and mixotrophic conditions. Assimilation kinetics of acetate and metabolite formation by *C. reinhardtii* cells, after twenty four hours of (A) mixotrophy and (B) heterotrophy following the addition of [1, 2-^13^C]-acetate to the TP medium containing CCCP (20 µM). Cells from different time points were pelleted, re-suspended in TP medium, followed by recording 1D proton decoupled [^13^C]-NMR spectra. (C) Control heterotrophic cells without CCCP, but processed similarly. (The signs * and ** represent methyl and carboxyl peaks, respectively, of [1, 2-^13^C]-acetic acid, and the signs^+^and^++^represent bicarbonate and CO_2_^aq^ peaks, respectively).

To probe the functional role of chloroplast and mitochondrial compartments in the metabolic assimilation of acetate, we used specific inhibitors. When the cells in mixotrophy were treated with atrazine [a photosystem-II (PS-II) inhibitor], photosynthesis got affected resulting in accumulation of bicarbonate and traces of CO_2_^aq^ similar to heterotrophic conditions (Fig. 4B) [15]. The mixotrophic state with PS-II inhibition resembled that of the heterotrophy to some extent. This was evident in a comparison, where cells in true heterotrophy showed higher levels of CO_2_^aq^ as compared to mixotrophic cells subjected to atrazine treatment (Fig. 4A and 4B). In contrast, when the cells were treated with carbonyl cyanide m-chlorophenyl hydrazone (CCCP), a mitochondria and chloroplast un-coupler of membrane potential in *C. reinhardtii*, neither bicarbonate nor CO_2_^aq^ was formed in heterotrophic or mixotrophic cultures (Fig. 5A and 5B) [16]. CCCP being a general membrane uncoupler leads to drop in cellular ATP production, thereby seriously affecting cellular energy status. CCCP treated cells showed only [1, 2-^13^C]-acetate signatures. To find out whether these signatures come from the acetate in the medium as a consequence of acetate uptake inhibition, if any, we pelleted the cells, resuspended and measured ^13^C-acetate signatures (if any). Acetate uptake was evidenced with the observation of ^13^C-acetate signatures in the NMR spectrum of the pelleted cells (Fig. 6A and 6B). It appeared that CCCP treated cells were able to assimilate acetate in both mixotrophic and heterotrophic states, but were unable to metabolize it further, perhaps due to energy imbalance caused by CCCP treatment. In the control cells, where the CCCP was not added, the cell pellet did show the expected metabolites, as mentioned earlier, namely, bicarbonate and CO_2_^aq^ (Fig. 6C). This experiment did uncover an important result that cellular ATP supply was essential for the metabolic assimilation, but not for the uptake of acetate during both mixotrophy and heterotrophy. These experiments involving specific inhibitors revealed that active photosynthesis and mitochondrial functions are essential for metabolic assimilation of acetate in both mixotrophic and heterotrophic states.

### Transition in ^13^C-metabolites following prolonged incubation of cells

*C. reinhardtii* cells are adept in surviving through long periods of nutritional stress by showing metabolic readjustments [1]. In order to assess such metabolic transitions, we analyzed samples beyond 24 hrs of incubation up to several days without supplementing them with any fresh medium.

**Figure 7:**
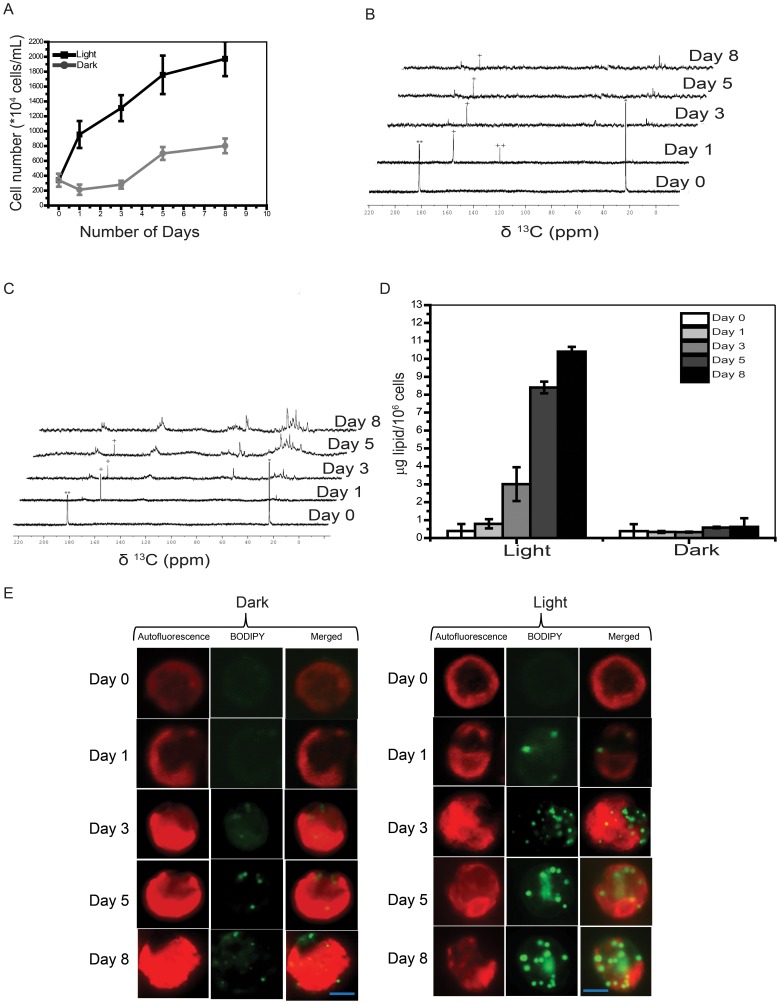
[1, 2-^13^C]-acetate metabolism monitored by NMR for eight days shows TAG accumulation in cells: a starvation response. Bright field counts of cells in mixo- *versus* heterotrophic incubation conditions over a period of eight days. Mean/SD were retrieved from three independent experimental measurements. (B–C) Assimilation kinetics of acetate by *C. reinhardtii* followed by TAG accumulation in the cells, as studied by recording proton decoupled 1D [^13^C]-NMR spectra, during (B) heterotrophy and (C) mixotrophy, at different time points. The spectra were recorded over a period of eight days, following the addition of [1, 2-^13^C]-acetate to the TP medium. (D) Total lipid estimation by phosphorvanillin assay of cells in mixo- *versus* heterotrophic incubation conditions over a period of eight days. Values are mean of three independent experiments ± SD. (E) Confocal image of lipid body formation due to TAG accumulation detected by BODIPY staining and chloroplast autofluorescence of the cells on different days of growth in mixotrophy and heterotrophy. Bar corresponds to 5µm. (The signs *, ** represent methyl and carboxyl peaks of [1, 2-^13^C]-acetic acid, and the signs^+^and^++^represent bicarbonate and CO_2_, respectively).

Cell count analyses as a function of incubation for the period beyond 24 hrs revealed steady rise in cell number during mixotrophy while the growth was much slower during heterotrophy (Fig. 7A). As described earlier, by the end of first day most of labeled acetate was converted to bicarbonate (in mixo/heterotrophy) and CO_2_^aq^ (in heterotrophy) (Fig. 7B and 7C). Further incubation beyond the first day revealed the following transitions. On the third day, we noticed several spectral signatures arising from lipid in mixotrophic culture (Fig. 7C). In contrast, heterotrophic cells exhibited much reduced levels of lipid accumulation (Fig. 7B). Biochemical estimation of total lipid content corroborated the result where mixotrophic culture showed much higher accumulation of lipid as compared to heterotrophic culture (Fig. 7D). Comparison with the standard ^13^C-NMR spectrum of glyceryltrioleate matched the spectral signatures of TAG in both mixo- and heterotrophic cells (Fig. 7B, C, S5C and D). Accumulation of lipid signals was concomitantly associated with drop in bicarbonate and CO_2_^aq^ levels (Fig. 7B and 7C). Most of the carbon flux was routed towards lipid body production.

We corroborated lipid accumulation in these nutritionally stressed cells, by staining them with BODIPY that ascertained lipid-body accumulation. Live cell staining with BODIPY revealed that cells grown in mixotrophic state for five and eight days accumulated several lipid bodies (Fig. 7E). Lipid body formation has been demonstrated earlier in cells undergoing nutrient stress due to either nitrogen or sulphur starvation [17], [18]. Auto fluorescence imaging showed cup-shaped chloroplasts in these cells. In comparison, heterotrophic cells showed fewer and smaller lipid bodies compared to mixotrophic cells (Fig. 7E). BODIPY stained lipid body signal levels correlated well with the respective NMR spectral signatures associated with TAG (Fig. 7B, 7C & 7E).

All these experimental observations taken together revealed that *C. reinhardtii* cells exhibit a rapid time dependent uptake of acetate well within the first 24 hrs, followed by relatively slow metabolic conversion to bicarbonate and CO_2_^aq^, which in turn got further metabolized rather slowly into starch and lipids during prolonged conditions of incubation and nutritional starvation.

### Metabolic changes associated with photoautotrophic assimilation of ^13^C- bicarbonate

**Figure 8:**
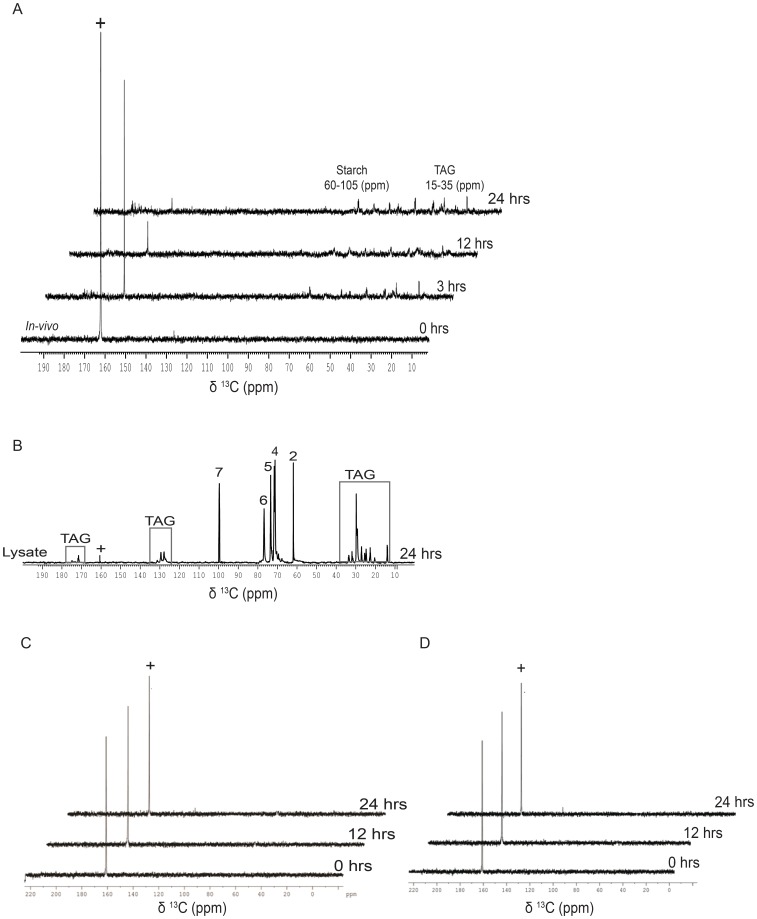
Assimilation kinetics of [^13^C]-Sodium bicarbonate during autotrophy in *C. reinhardtii*. (A) Assimilation kinetics of *C. reinhardtii*, as studied by recording proton decoupled 1D [^13^C]-NMR spectra during the light phase of growth (photoautotrophy) using [^13^C]-bicarbonate. (B) [^13^C]-NMR cell lysate spectrum of cells incubated in light for 24 hrs. (C) Assimilation kinetics of [^13^C]-bicarbonate by *C. reinhardtii*, as studied by recording proton decoupled 1D [^13^C]-NMR spectra, during the dark incubation control, and (D) atrazine treated cells in the light phase of growth (negative control of autotrophy) at different time points, following the addition of the 20 mM [^13^C]- bicarbonate to the TP growth medium.

Efficient uptake of organic carbon, followed by its metabolic breakdown led to the accumulation of inorganic carbon, namely bicarbonate and CO_2_^aq^ in the heterotrophic cells. In order to contrast the same when bicarbonate was used as a sole source of carbon, we performed experiments by feeding the cells with ^13^C-bicarbonate. Cells from a mixotrophically grown exponential culture were pelleted down, resuspended in TP-medium containing ^13^C-bicarbonate (Fig. S1), and followed it by NMR analyses of time-dependent changes in metabolites, *in-vivo*. As expected, uptake of the labeled bicarbonate was efficient in light phase (photoautotrophic) cultures (Fig. 8A). Photoautotrophically grown cells when fed with 20 mM ^13^C-sodium bicarbonate in the presence of light showed the decline of bicarbonate peak at 161.01 ppm, accompanied with the appearance of starch and lipid signatures with in the 24 hrs regime (Fig. 8A). To enhance the S/N ratio of starch and lipid signals, we sonicated the cells and recorded the ^13^C-NMR spectrum with the lysate (Fig. 8B). This resulted in a dramatic enhancement of the S/N ratio. We recovered convincing signatures of starch signals (numbered as 2, 4, 5, 6 and 7 in Fig. 8B) and that of TAG (boxed spectral lines in Fig. 8B). When the cells were fed with ^13^C-acetate, such early appearance of starch and lipid signals were not noticed (Fig. 3A with 8A). The ^13^C-bicarbonate uptake failed when the cells were incubated in dark (Fig. 8C). Furthermore, when atrazine was added during light incubation in bicarbonate feeding experiment, neither the uptake nor the metabolite formation was seen (Fig. 8D). As expected, this result was similar to cells that were fed with bicarbonate in dark (Fig. 8C). Photosynthetic assimilation of Ci was the main driver for Ci uptake from the medium. In the following paragraphs, we discussed all these *in-vivo* results and summarized the main conclusions in the current context.

## Discussion

We employed *in-vivo* NMR to study cellular metabolism in *Chlamydomonas reinhardtii* using [1, 2-^13^C]-acetate as the sole carbon source and compared the same in heterotrophy *versus* mixotrophy, as a function of time. The assimilatory kinetics in heterotrophy resulted in near complete uptake of 20 mM acetate by the cells from the medium within the first 12 hrs. On the other hand, the kinetics were slower in mixotrophic culture as compared to heterotrophic culture under identical growth conditions. This is primarily due to the competing entry of carbon via CO_2_ fixation during mixotrophic growth (Fig. 1B and 3B) [1]. Heterotrophic growth revealed an accumulation of two predominant metabolites, namely bicarbonate and CO_2_^aq^, with concomitant acetate uptake. Interestingly, the steady state level of intracellular Ci scaled linearly with the time of acetate incubation in heterotrophy (Fig. S6), suggesting that the culture conditions maintained a steady state in the carbon flux, at least within that time duration. Mixotrophic growth revealed largely the buildup of bicarbonate in the cells but not CO_2_^aq^, as the cells fix it photosynthetically. The CO_2_^aq^ formed through the TCA cycle in the mitochondria gets converted to bicarbonate by multiple carbonic anhydrases present in *C. reinhardtii* cell [3]. This metabolism continues unhindered both in the dark and light phases of growth. As expected, both carbons of acetate contributed to CO_2_^aq^ formation during heterotrophy (Fig. 2A and 2B). During TCA cycle assimilation of acetate, if one follows ^13^C-label at 1, 2-carbons of citrate, conversion of alpha-keto glutarate to succinyl-CoA leads to the release of ^13^C-label from carboxyl carbon as ^13^CO_2_. The second ^13^C (from methyl group of acetate) ends up labeling the terminal-C in oxaloacetate, which is released as ^13^CO_2_ in the next round of TCA-cycle. ^13^C-carbons at 5, 6 locations of citrate can undergo similar fate akin to that of 1, 2 carbons, due to intrinsic symmetry in citrate backbone, thereby leading to reduced contribution of methyl carbon towards ^13^CO_2_ as compared to carboxyl carbon (Fig. S6). The asymmetry of carboxyl *versus* methyl carbon flow of acetate during TCA cycle, as described above, explained the higher rate of contribution by carboxyl *versus* methyl carbon to the net Ci yield per acetate *in-vivo*. Ashworth et al have similarly shown the expected chemical asymmetry in the routing of acetate carbons towards the carbon-skeleton in triacylglycerol in corn cells [19]. Unlike bicarbonate, CO_2_^aq^ does not diffuse out of cells and gets accumulated in the dark (Fig. S4). For NMR to detect CO_2_ levels so prominently in heterotrophic cells, CO_2_^aq^ level should have reached close to millimolar concentrations in the cells. Interestingly, we note that high level of CO_2_^aq^ detected in dark showed no NMR signature of bound CO_2_. While bicarbonate and CO_2_ were detectable by normal NMR analyses, we had to employ high-sensitive TXO probe to detect low abundance carbon metabolites formed in the heterotrophic and mixotrophic cells within the first 24 hrs. The 1D ^13^C-spectra recorded with the TXO probe revealed the spectral signatures of starch and glycerol as additional ^13^C-metabolites (Fig. 1C). In contrast, both starch and TAG signatures were evident in NMR analyses for cells grown in mixotrophic conditions (Fig. 3C). Accumulation of glycerol in heterotrophic cells suggested that fatty acid acylation of glycerol was reduced, thereby affecting TAG formation in dark as compared to mixotrophic cells (Fig. 1C *versus* 3C). It is known that fatty acid acylation of glycerol in plants depends on light conditions, which is why TAG levels were low in dark (Fig. 7B) [20]. Accumulation of TAG was highly photosynthesis dependent (Fig. 7C) and inhibition of PSII by 3-(3,4 dichlorophenyl)-1,1-dimethylurea (DCMU) led to abolition of TAG production in the cells [21]. Here, we also make a note of how bicarbonate-feeding experiment contrasted with that of acetate, both in dark and light incubation conditions. Photoautotrophically grown cells when fed with [^13^C]- bicarbonate in the presence of light showed uptake of bicarbonate, accompanied by the appearance of starch and lipid signatures within the first 24 hrs (Fig. 8A and 8B). Importantly, when the cells were fed with labeled acetate, we had not seen such early appearance of starch and lipid signals in dark (heterotrophy) or even in light (mixotrophy) by normal detection conditions of NMR (Compare Fig. 1A, 3A with 8A). The starch and lipid signals could be captured only with high-sensitive TXO probe (Fig. 1C and 3C). Early production of starch and lipid during photoautotrophy was suggestive of a more efficient flux of carbon towards the starch and lipid biogenesis via Calvin cycle route. Even though acetate assimilation during mixotrophy leads to starch and lipid accumulation, it takes much longer time. Several days of nutritional stress precedes starch and lipid formation during mixotrophy. We suggest that this delay is perhaps due to the conditions of high intracellular Ci and attendant low efficiency of carbon concentrating mechanism (CCM) that eventually slows down the production of carbon-rich products such as starch and lipid [22] (Fig. S7). Photoautotrophy at air-level CO_2_ induces high CCM catalyzing early production of starch and lipid chains in the cells (Fig. 8A & S7). Time course analyses revealed that cells in hetero/mixotrophy mandatorily proceed via inorganic carbon pathway (bicarbonate & CO_2_^aq^) towards starch and lipid biosynthesis and not directly via gluconeogeneic route of acetate assimilation.

*C. reinhardtii* cells enter into exponential growth phase after about 4–5 days following inoculation and sustain long stationery phase spanning several days (∼6–7 days), when the C/N nutrients in the medium are not supplemented [23]. Only following a highly regulated starvation phase response, involving starch and TAG accumulation spanning several days during stationery phase, do the cells enter into cell death phase [17]. Therefore *C. reinhardtii* cells offer an excellent system to study the metabolic transitions that ensue during the stationery phase, where the cells are divisionally inactive, but metabolically still dynamic. The Ci metabolites get efficiently channeled towards TAG, which reflected a classical remodeling of metabolism towards achieving long-term lipid storage in the stationery phase cells (Fig. 7C).

All the effects described above must have been due to active functioning of mitochondria and chloroplast in the cells. Inhibition of either or both organellar functions had consequences on the yield of the observed metabolites. Inhibition by atrazine, arrested PSII function, rendering mixotrophic culture akin to that of heterotrophic state: acetate uptake led to the intracellular buildup of CO_2_^aq^ even in light-grown cells (Fig. 4A and 4B); bicarbonate assimilation failed in the presence of light during photo-autotrophy, again akin to the conditions of heterotrophy (Fig. 8C). Mitochondrial and chloroplast activity inhibition, achieved by treating the cells with CCCP uncoupler, led to the arrest of acetate metabolism during both mixotrophy and heterotrophy even though cells were proficient in acetate uptake (Fig. 5 and 6). It appears that active functioning of mitochondria and chloroplast and collaboration between them is crucial for coordinating the carbon flux within the cells. Our current understanding of acetate assimilation in mixotrophy *versus* heterotrophy is limited to the extent of specifying the pathways associated with it, namely, the production of acetyl-CoA that further metabolizes through either glyoxylic acid cycle, tricarboxylic acid cycle, pentose phosphate cycle or gluconeogenesis [24]. An excellent review by Perez-Garcia recently summarized a comparative account of different algal species assimilating acetate or other organic acids [5, 25 and 26]. However, none of the common predominant metabolites of glyoxylic or TCA cycle were detectable by NMR analyses performed in the current study. This is either due to poor sensitivity level of NMR or low abundance of these intermediates due to relatively high flux rates of the associated pathways or both. In fact one of the current exciting challenges in the area of metabolism is to quantify the relative flux rates of various competing pathways in order to rationalize the plasticity associated with cellular physiology. Recent flux balance analyses reported on *C. reinhardtii* metabolism and computed relative culture growth rates with respect to carbon efficiency [27]. Our understanding is still far from being able to dissect relative contributions of flux rates of various metabolic crisscrossing pathways during different culture conditions. Moreover, detailed studies are required to map the metabolic coupling mechanisms that operate between organelles, such as chloroplast, mitochondria in *C. reinhardtii* cells. Combination of *in-vivo* analyses with high throughput metabolic studies as a function of time during heterotropy *versus* mixotrophy would provide the required insights.

## Materials and Methods

### Growth conditions for NMR measurements

*C. reinhardtii* [CC-125 wild-type mt+] cells were grown at 25°C in Tris acetate phosphate (TAP; pH 7.0) buffered medium [1]. The cells were exposed to a light flux of 135 µmol. photons m^−2^ s^–1^ from the bottom of a shaker, set at 200 rpm. The cells were grown in unlabeled TAP from a 1% primary inoculum in a continuous light till the culture reached the end of log phase (∼4 to 5 million cells per mL) growth, following which the cells were washed thrice in the TP medium (TAP without acetate). Following this the cells were suspended in a fresh TP medium containing 20 mM [1, 2-^13^C]- sodium acetate or [1-^13^C]−/[2-^13^C]-sodium acetate or [^13^C]-sodium bicarbonate (99% Cambridge Isotope Laboratories, Cat no. CLM-440-1, CLM-441-5) and incubated in either continuous light or dark conditions for a specified length of time, during which aliquots of 0.5 mL were withdrawn at various time intervals for NMR measurements (Fig. S1). For NMR experiments with cell-lysate, the lysate was prepared by sonication of the cells at 15 s pulse of 40% amplitude. For inhibitor experiments, the cells were treated as above in the presence or absence of atrazine (5 µM) and carbonyl cyanidem-chlorophenyl hydrazone-CCCP (20 µM). Cell concentrations were determined by using a Neubauer Hemocytometer cell counting chamber (Cat. no. 100 Hausser Scientific Blue Bell Pa. USA).

### NMR measurements

Basic background: NMR is used as a technique to discern the nature of chemical changes happening in the culture medium as well as in cells and cell lysates. It is a versatile analytical tool where NMR spectral readouts depict the nature of chemical species identified by their unique chemical shifts expressed in ppm (parts per million).

NMR experiments were on a Bruker Avance 800 MHz NMR spectrometer, equipped with a cryogenically cooled 5 mm triple-resonance probe. For each of the sample retrieved from the culture at different time points of acetate or bicarbonate assimilation, a proton decoupled 1D ^13^C-NMR spectrum was recorded at 25°C, with the same set of acquisition parameters. The pulse program ‘zgdc’ was used with the ^1^H-carrier placed on H_2_O resonance (4.68 ppm) and ^13^C-carrier at 100 ppm. All the spectra were recorded with a ^13^C-pulse width of 12 µs corresponding to a 90° flip angle, an acquisition time of 0.164 s, a relaxation delay of 50 s (>5×T_1_, to achieve a complete relaxation of the spin systems before acquiring each of the subsequent scan; The T_1_ values for the methyl and carboxylic ^13^C nuclei measured on an 800 MHz NMR spectrometer, at 25°C, were 6.9 and 8.5 s, respectively (data not shown)), 16384 acquisition data points, and 8 scans. Proton decoupling was kept ‘on’ during ^13^C-NMR detection. Total acquisition time for each sample was ∼10 min, during which time, the sample does go through an unavoidable dark incubation step at room temperature (25°C; Fig. S1). After every NMR data collection, the cells retrieved from NMR tube were found alive and active as assessed by cell mobility and O_2_-uptake assays, suggesting that the study reflects real-time analyses of acetate metabolism in cells as the samples were withdrawn from an active culture and put through NMR acquisition only for 10 min. The spectral signatures of predominant metabolites detected do not get affected during such short NMR data acquisition time as the metabolic changes occur at much slower rates. All 1D data was processed with the same processing parameters by the standard protocol provided by the spectrometer vendor. In the case of doublet signatures, the area under both the components was integrated and added up. All chemical shifts were referenced with an addition of standard dioxane, which shows its lone ^13^C signature at 67.4 ppm. The ^13^C signals of tris buffer arising from CH_2_-OH (at 61.89 ppm) and the quaternary carbon (at 59.38 ppm) were noticed in the spectrum. Some 1D ^13^C-NMR spectra were recorded with ‘zgig’ pulse program on a Bruker Avance 700 MHz NMR spectrometer equipped with a TXO probe, specifically designed for ^13^C/^15^N direct detection with enhanced signal-to-noise ratio. The acquisition parameters in these experiments were the following: a ^13^C-pulse width of 10.3 µs corresponding to a 90° flip angle, an acquisition time of 0.186 s, a relaxation delay of 2 s, 16384 acquisition data points, and 512 scans. All the data were processed with a 10 Hz line-broadening parameter. All NMR spectra were corroborated by a minimum of three independent biological experiments. Representative spectra are shown for only one but quantitation was based on data from triple repeats. The spectra from the same experiment are shown in the form of a standard *“stacked plot”*, so that the equivalent peaks appear non-overlapping and shifted for better visualization. The NMR data representation is similar to that in previous studies [28]–[33]. Typically, all NMR data are quantified by spectral line integration. All the quantitative data described in the current study has been retrieved from at least three independent NMR experiments from where mean and SD was calculated.

### Measurement of Inorganic sodium bicarbonate species at different pH

^13^C- NMR was used to observe inorganic ^13^CO_2_^aq^ species by subjecting a 10 mM solution of ^13^C-sodium bicarbonate to varying pH. At pH 7 the ^13^C-spectra showed a single peak at 161.01 ppm. When the pH was lowered to two, with the addition of 1 M HCl, only the ^13^CO_2_^aq^ spectral signature was seen in the spectrum at 125.48 ppm. When the pH was increased to a value of 10 by the addition of 1 M NaOH a single peak was observed at 164.71 ppm, due to fast equilibration kinetics of HCO_3_^−^/CO_3_^2−^ anions depending on their relative concentrations. However, when pH is further increased to 10.5, a single peak at 168.42 ppm representing CO_3_^2−^ species started appearing (Fig. S3C).

### BODIPY staining of lipid bodies in *C. reinhardtii*

A lipophilic fluorescent dye 4,4-difluoro-1, 3, 5, 7, 8-pentamethyl-4-bora-3a, 4a-diaza-s-indacene (BODIPY; 493/503; Cat no D3922 Molecular probes, Life technologies Eugene OR, USA) was used for detecting *C. reinhardtii* lipid body formation, as described by Work et al [34]. *C. reinhardtii* cells pelleted down after centrifugation were resuspended in a total volume of 100 µL containing 5 µg/mL BODIPY, taken from a stock solution of the BODIPY (10 mg/mL of it in dimethyl-sulfoxide). The concentration of the cells was around 1 million per 100 µL. Such cells were incubated in dark for 10 min. Following the incubation the cells were washed thrice in TP medium and embedded in 1% molten low-melting agarose kept at around 37°C, in order to capture the cells in a fixed plane for imaging. The cell suspension and the molten agarose were taken in 1∶1 concentration ratio. A cover slip was placed over such agarose-cell suspension before it could solidify. Following this, confocal images were acquired using a Zeiss LSM 510 meta confocal microscope. The output power of the laser used in the setup was 7 mW, with an emission wavelength of 488±0.5 nM. Chlorophyll auto-fluorescence was detected using a 685/70 band-pass optical filter, and BODIPY (493/503) fluorescence was detected using a 515/30 band-pass optical filter.

### Lipid estimation

Lipid estimation in the cell cultures (under different incubations and at different time points) of acetate assimilation was carried out using the classical sulpho-phosphor-vanillin assay (SPVA), as described by Cheng et al [35]. Lipid extraction from different cell cultures was carried out by adopting the methonal-chloroform method of extraction [36]. The lipid samples thus extracted were diluted such that the sample concentration was within the range of the detection of the SPVA assay. An amount of 100 µL each of above-mentioned lipid extracts were placed at the bottom of wells in a microplate, and the solvent was evaporated by incubating the microplate at 90°C. Following this, 100 µL of concentrated sulfuric acid was added to each of the well and the microplate was then incubated again at 90°C for 20 min. The microplate was cooled on an ice-bath and background absorbance was measured at 540 nm. Vanillin–phosphoric acid reagent (50 µL; 0.2 mg/ml vanillin taken in 17% phosphoric acid) was added to each well for the development of color. The absorbance was read at 540 nm after 10 min. The lipid concentrations were determined using glyceryltrioleate as a standard.

## Supporting Information

Figure S1 **Experimental scheme.**
(TIF)

Figure S2 **Proton decoupled 1D [^13^C]-NMR spectrum of [1, 2-^13^C]-acetate.**
(TIF)

Figure S3 **Proton decoupled 1D [^13^C]-NMR spectra of (A) [^13^C]-bicarbonate dissolved in water, (B) CO_2_^aq^** **(dry-ice) in water, and (C) [^13^C]-bicarbonate at different pH values (2.5 to 12).**
(TIF)

Figure S4 **Assimilation kinetics of [1, 2-^13^C]-acetate in the cell-free supernatant and cell-pellet of** ***C. reinhardtii*** **culture cells from heterotrophic state.** Assimilation kinetics of acetate in *C. reinhardtii* during heterotrophic incubation, as studied by recording proton decoupled 1D [^13^C]-NMR spectra of samples taken from different time points, after adding the [1, 2-^13^C]-acetate to the TP medium. Proton decoupled [^13^C]-NMR spectra of (A) cell-free supernatant (B) cell pellet re-suspended in TP medium. (The signs * and ** represent methyl and carboxyl peaks, respectively of [1, 2-^13^C]-acetic acid, and the signs^+^and^++^represent bicarbonate and CO_2_^aq^ peaks, respectively).
(TIF)

Figure S5 **Chemical shift assignment by standard glycerol, starch and TAG.** (A) Proton decoupled 1D [^13^C]-NMR spectrum of pure glycerol. (B) Proton decoupled 1D [^13^C]-NMR spectrum of wheat starch as assessed by solution NMR. (C) Proton decoupled 1D [^13^C]-NMR spectrum of TAG on the 8^th^ day of mixotrophic culture sample (D) Assigned chemical shifts of standard glyceryltrioleate (TAG).
(TIF)

Figure S6 **Comparison of the rate of Ci release per acetate assimilation from methyl** ***versus*** **carboxyl [^13^C] carbons in acetate during heterotrophy.** Total Ci (bicarbonate plus CO_2_ signals) in Fig. 2A and 2B in each spectrum was normalized to labeled acetate signal and plotted as a function of time. Values are mean of three independent experiments ± SD.
(TIF)

Figure S7 **A model summarizing photoautotrophic** ***versus*** **mixotrophic modes of carbon assimilation in** ***C. reinhardtii*** **.** We propose that high *versus* low CCM efficiency in photoautotrophic *versus* mixotrophic culture, respectively, leads to differential kinetics of starch and lipid accumulation via inorganic carbon pathway.
(TIF)
