# Supplementary figures and images for: Acetate and Bicarbonate Assimilation and Metabolite Formation in Chlamydomonas reinhardtii: A 13C-NMR Study
Source: PLoS One. 2014 Sep 10;9(9):e106457. doi: 10.1371/journal.pone.0106457 (PMC4160175; doi:10.1371/journal.pone.0106457)

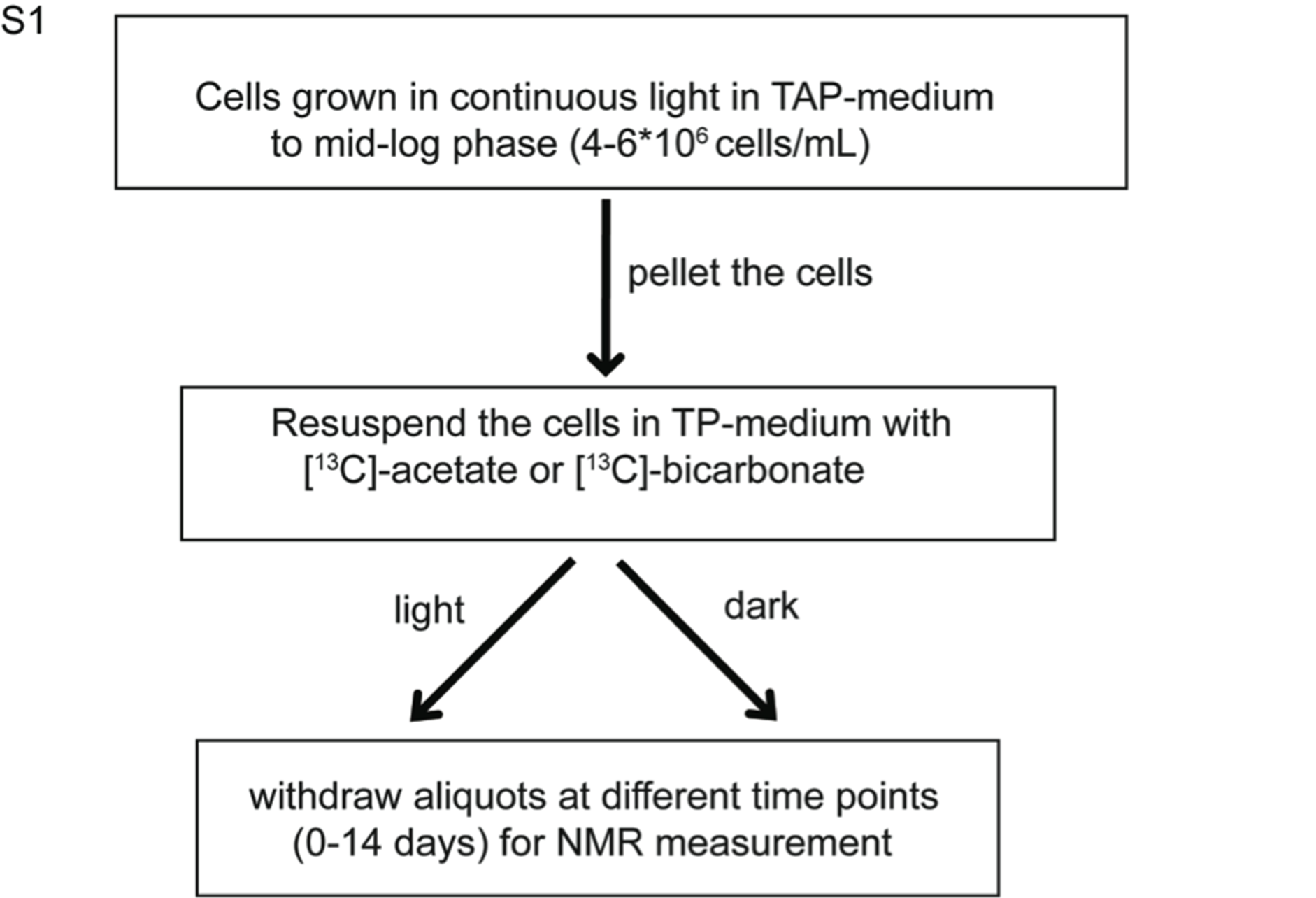

Supplement: Figure S1 — Experimental scheme. (TIF) [file pone.0106457.s001.tif]

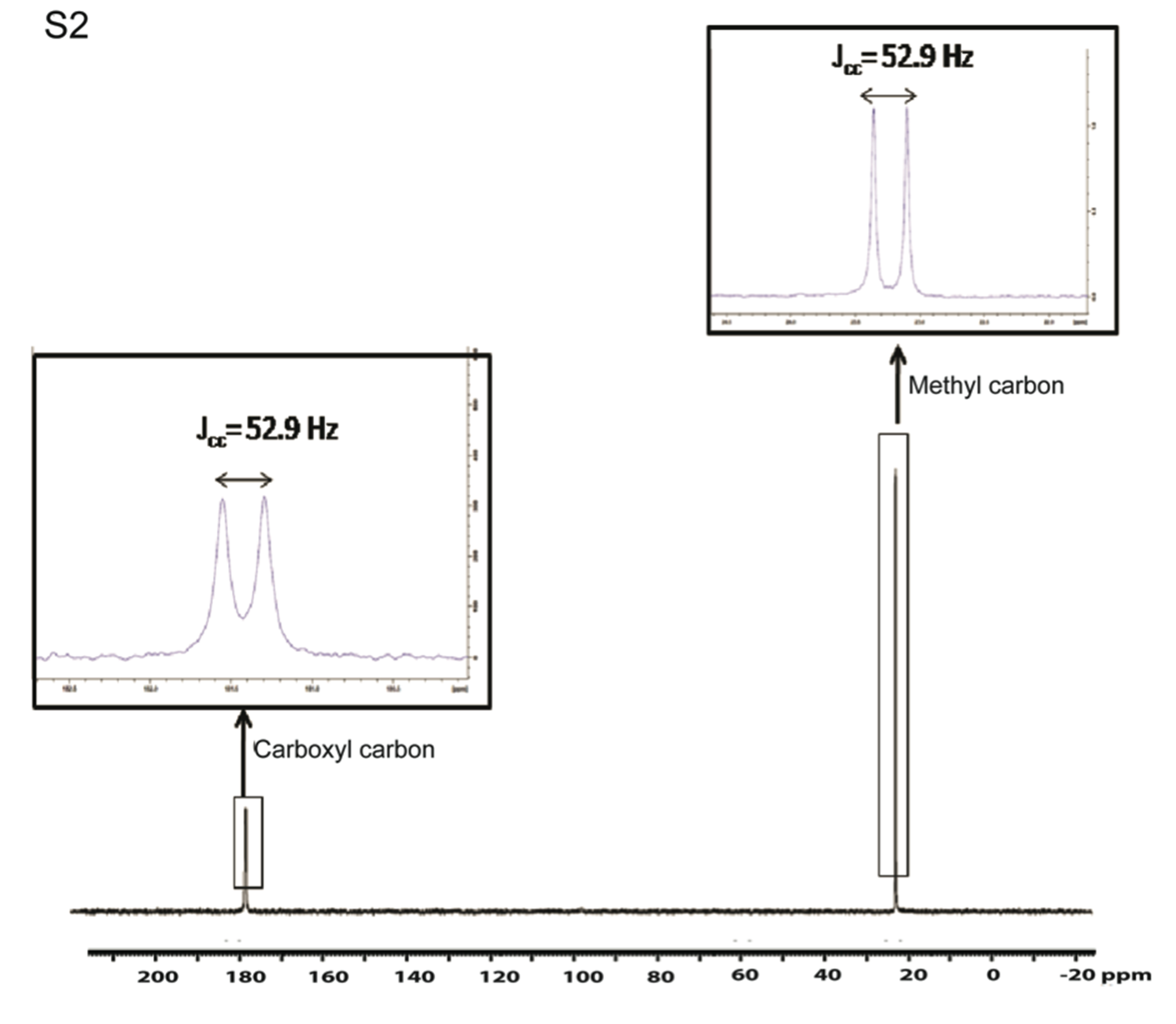

Supplement: Figure S2 — Proton decoupled 1D [13C]-NMR spectrum of [1, 2-13C]-acetate. (TIF) [file pone.0106457.s002.tif]

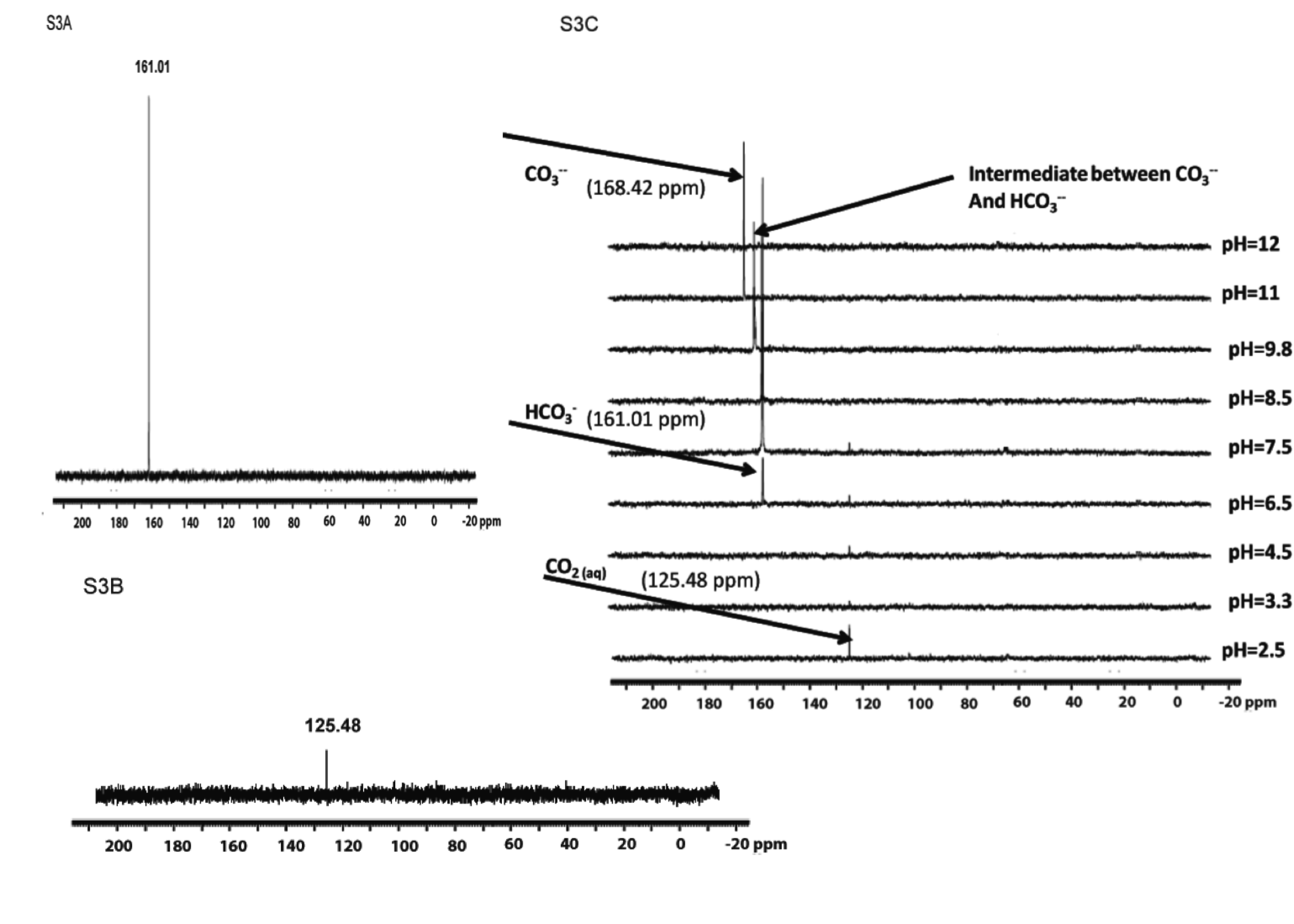

Supplement: Figure S3 — Proton decoupled 1D [13C]-NMR spectra of (A) [13C]-bicarbonate dissolved in water, (B) CO2aq (dry-ice) in water, and (C) [13C]-bicarbonate at different pH values (2.5 to 12). (TIF) [file pone.0106457.s003.tif]

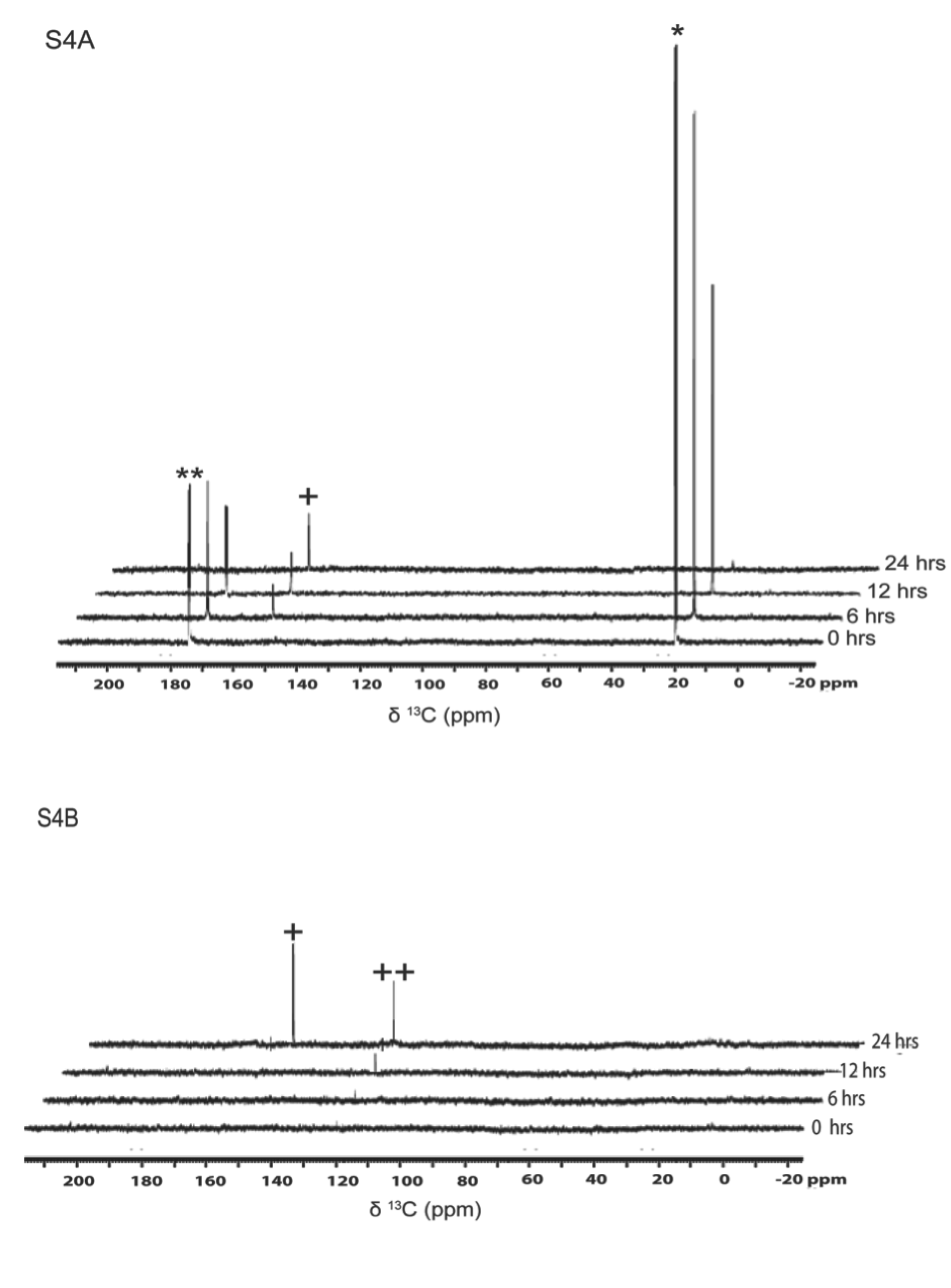

Supplement: Figure S4 — Assimilation kinetics of [1, 2-13C]-acetate in the cell-free supernatant and cell-pellet of C. reinhardtii culture cells from heterotrophic state. Assimilation kinetics of acetate in C. reinhardtii during heterotrophic incubation, as studied by recording proton decoupled 1D [13C]-NMR spectra of samples taken from different time points, after adding the [1, 2-13C]-acetate to the TP medium. Proton decoupled [13C]-NMR spectra of (A) cell-free supernatant (B) cell pellet re-suspended in TP medium. (The signs * and ** represent methyl and carboxyl peaks, respectively of [1, 2-13C]-acetic acid, and the signs+and++represent bicarbonate and CO2 aq peaks, respectively). (TIF) [file pone.0106457.s004.tif]

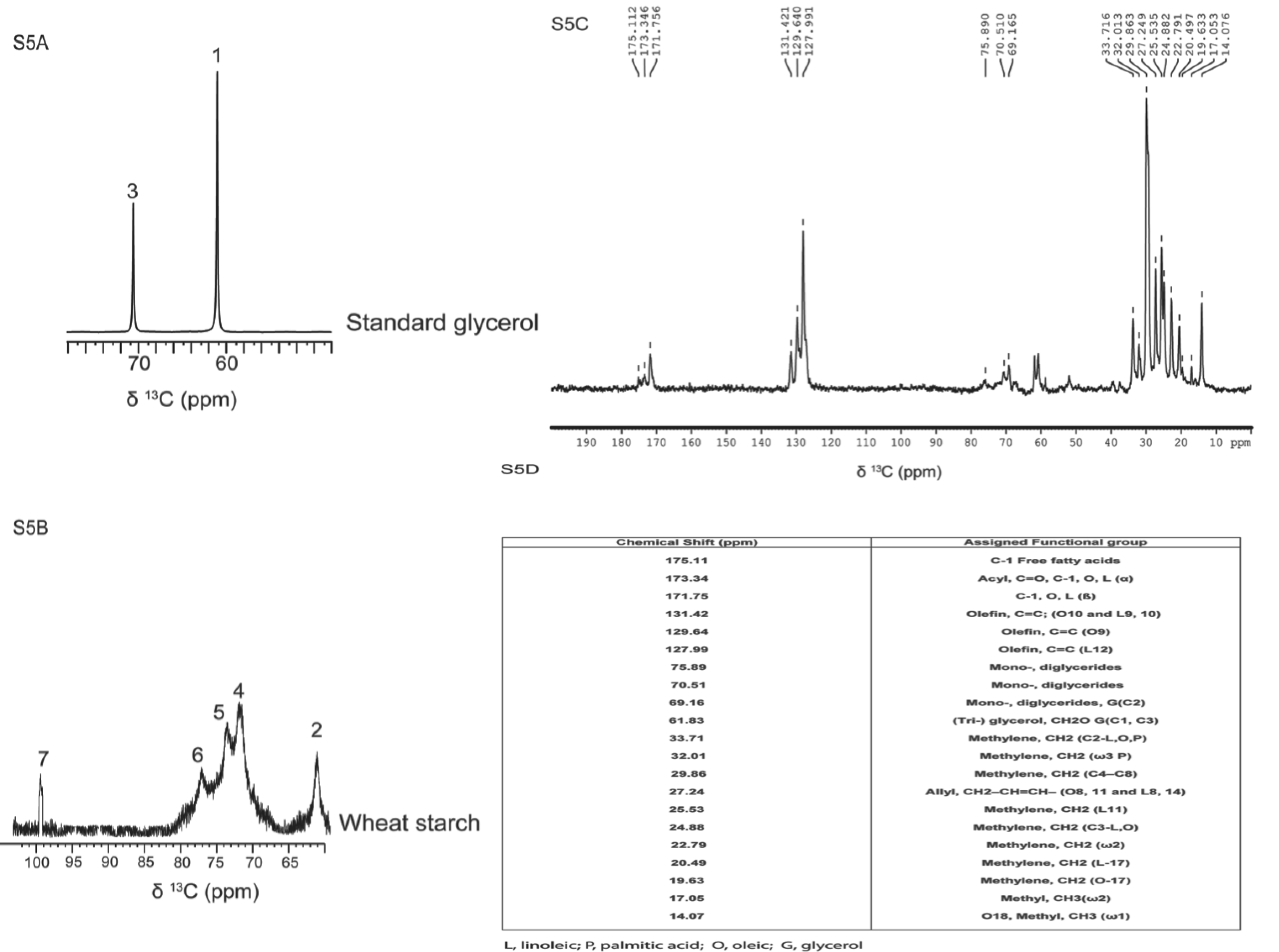

Supplement: Figure S5 — Chemical shift assignment by standard glycerol, starch and TAG. (A) Proton decoupled 1D [13C]-NMR spectrum of pure glycerol. (B) Proton decoupled 1D [13C]-NMR spectrum of wheat starch as assessed by solution NMR. (C) Proton decoupled 1D [13C]-NMR spectrum of TAG on the 8th day of mixotrophic culture sample (D) Assigned chemical shifts of standard glyceryltrioleate (TAG). (TIF) [file pone.0106457.s005.tif]

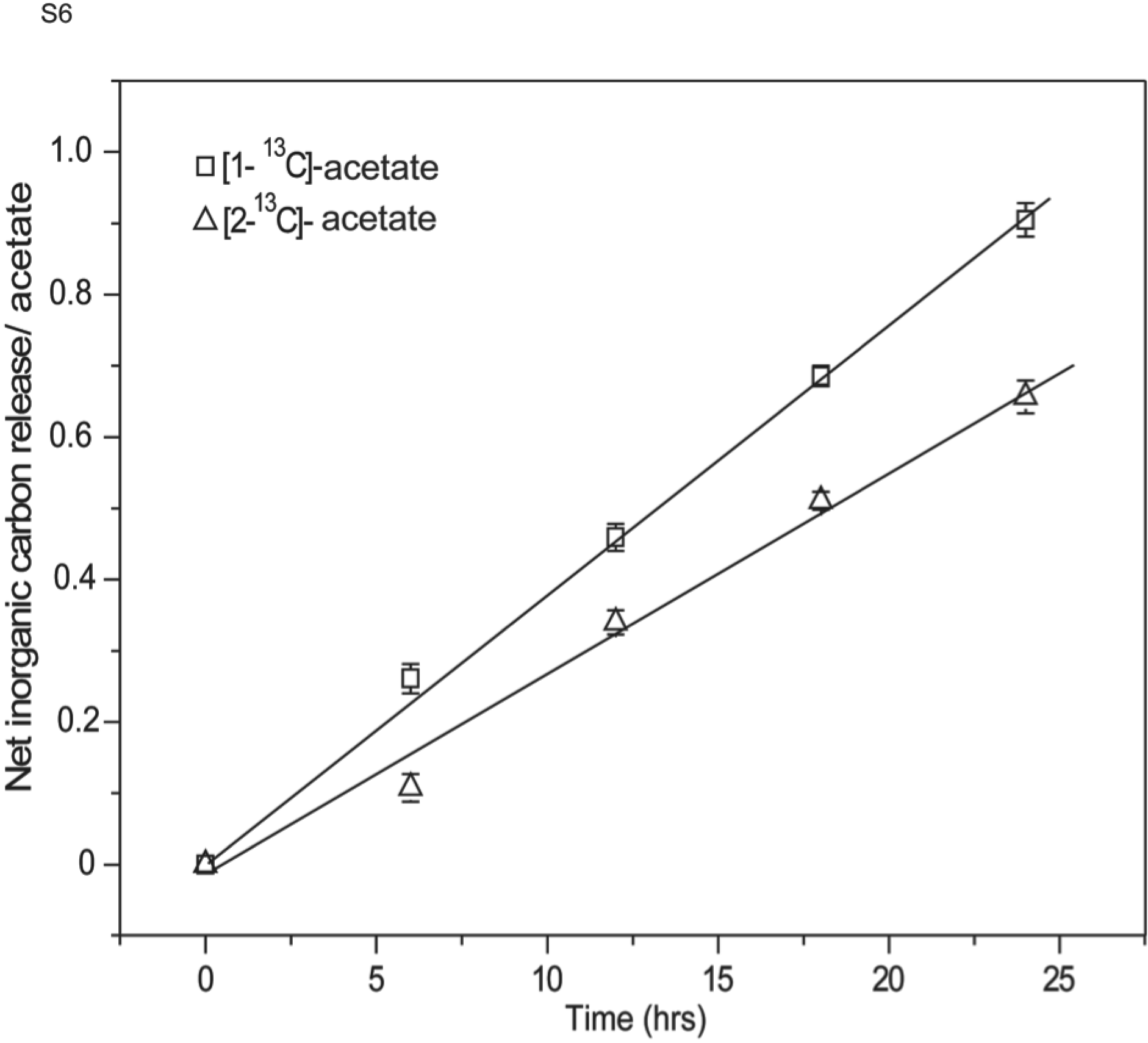

Supplement: Figure S6 — Comparison of the rate of Ci release per acetate assimilation from methyl versus carboxyl [13C] carbons in acetate during heterotrophy. Total Ci (bicarbonate plus CO2 signals) in Fig. 2A and 2B in each spectrum was normalized to labeled acetate signal and plotted as a function of time. Values are mean of three independent experiments ± SD. (TIF) [file pone.0106457.s006.tif]

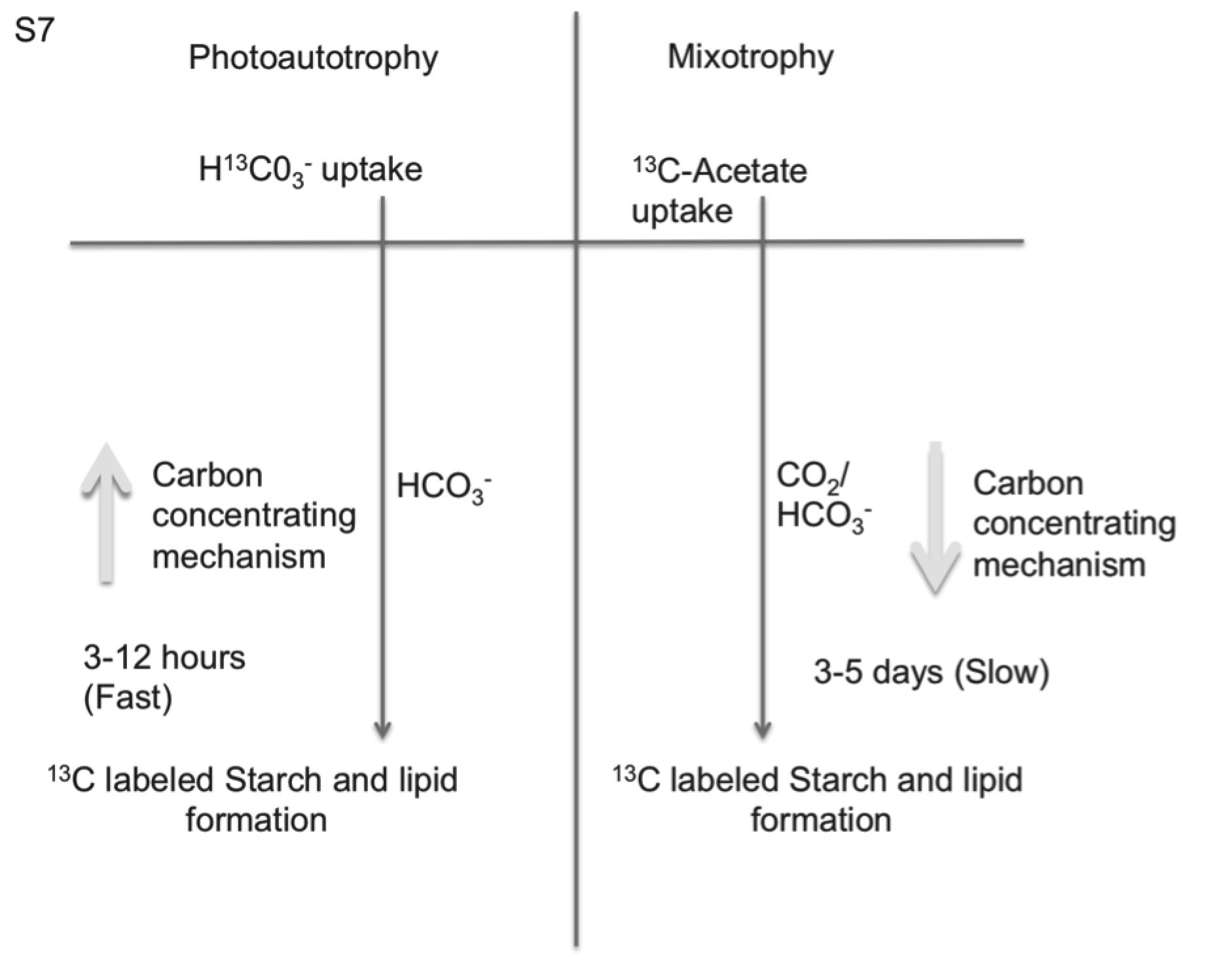

Supplement: Figure S7 — A model summarizing photoautotrophic versus mixotrophic modes of carbon assimilation in C. reinhardtii . We propose that high versus low CCM efficiency in photoautotrophic versus mixotrophic culture, respectively, leads to differential kinetics of starch and lipid accumulation via inorganic carbon pathway. (TIF) [file pone.0106457.s007.tif]
